# Room-temperature valley transistors for low-power neuromorphic computing

**DOI:** 10.1038/s41467-022-35396-x

**Published:** 2022-12-15

**Authors:** Jiewei Chen, Yue Zhou, Jianmin Yan, Jidong Liu, Lin Xu, Jingli Wang, Tianqing Wan, Yuhui He, Wenjing Zhang, Yang Chai

**Affiliations:** 1 Department of Applied Physics, The Hong Kong Polytechnic University, Hong Kong, China; 2 The Hong Kong Polytechnic University Shenzhen Research Institute, Shenzhen, China; 3 Wuhan National Laboratory for Optoelectronics, Huazhong University of Science and Technology, 430074 Wuhan, China; 4 International Collaborative Laboratory of 2D Materials for Optoelectronics Science and Technology, Shenzhen University, 518060 Shenzhen, China; 5 Frontier Institute of Chip and System, Fudan University, Shanghai, China

**Keywords:** Electronic devices, Electronic devices

## Abstract

Valley pseudospin is an electronic degree of freedom that promises highly efficient information processing applications. However, valley-polarized excitons usually have short pico-second lifetimes, which limits the room-temperature applicability of valleytronic devices. Here, we demonstrate room-temperature valley transistors that operate by generating free carrier valley polarization with a long lifetime. This is achieved by electrostatic manipulation of the non-trivial band topology of the Weyl semiconductor tellurium (Te). We observe valley-polarized diffusion lengths of more than 7 μm and fabricate valley transistors with an ON/OFF ratio of 10^5^ at room temperature. Moreover, we demonstrate an ion insertion/extraction device structure that enables 32 non-volatile memory states with high linearity and symmetry in the Te valley transistor. With ultralow power consumption (~fW valley contribution), we enable the inferring process of artificial neural networks, exhibiting potential for applications in low-power neuromorphic computing.

## Abstract (web summary)

Valleytronic devices employ the electronic valley degree of freedom to realize potential low-power electronic applications. Here, the authors utilize a topological semiconductor to engineer valley polarization transistors with long lifetimes and demonstrate low-power neuromorphic functionality at room temperature.

## Introduction

Charge-based electronics with conventional semiconductors are reaching their fundamental limits in power efficiency due to inevitable carrier scattering. Valley (a quantum degree of freedom of electrons) is an information carrier with high energy efficiency^1–3^, which is robust against low-energy phonons^4,5^, promising for next-generation electronics^6–8^. It still remains a grand challenge to realize room-temperature valley transistors, because the widely investigated excitonic valley has a short lifetime (<1 ps) due to long-range Coulomb interaction^4,6,9^. Room-temperature valley devices require the lifetime of valley polarization to be sufficiently long for propagating through the channel^9^. To achieve the valley polarization with a long lifetime^10^, researchers mainly adopted plasmonic hot-electron injection^3^ or interlayer excitation in van der Waals heterostructures^11^. However, it usually requires relatively complicated optical pumping designs and is incompatible with conventional field-effect transistors (FETs) modulated by the electric field.

Chiral anomaly in Weyl/Dirac materials allows to generate valley polarization that can diffuse over a long distance (~100 μm in theory) and sustain under thermal perturbation^12^, providing an alternative mechanism for room-temperature valley transistors (Supplementary Note 1). By applying an electric field that is parallel to the magnetic field, the valley polarization generated by the chiral anomaly can transport over a long distance at room temperature, because the relaxation process involves a large quasi-momentum transfer^12–14^. As the chiral anomaly-based valley transport arises from the non-trivial Berry curvature, we can realize valley transistors by modulating the strength of Berry curvature through the electrostatic gating at room temperature. By controlling the energy separation between Weyl point and Fermi level, the valley transistors can exhibit non-volatile multiple resistance states with high linearity and symmetry. Due to the robust valley transport against low-energy phonons^15^, the valley transistors have the potentials of realizing a high signal-to-noise ratio under low readout power, which are advantageous over conventional charge-based transistors with relatively large readout power ranging from nW to μW^16–18^. The valley transistors show the potentials for low-power neuromorphic computing, especially for the inference process.

In this work, we demonstrate room-temperature valley transistors with Weyl semiconductor tellurium (Te) with valley diffusion length over 7 μm. The valley transistors exhibit an ON/OFF ratio of 10^5^ through electrostatic gating. The ion insertion/extraction into the Te channel results in non-volatile valley resistance change with high linearity and symmetry, which allows to emulate artificial synaptic states. The robust valley transport characteristics enable to operate the synaptic valley devices with the ultralow readout voltage, allowing to construct an artificial neural network (ANN) with low power consumption (~fW valley contribution and ~pW Ohmic contribution) in the inferring process. This study provides a strategy for low-power neuromorphic computing with the low-loss valley devices.

## Results

### Valley transport of Weyl semiconductor

**Fig. 1:**
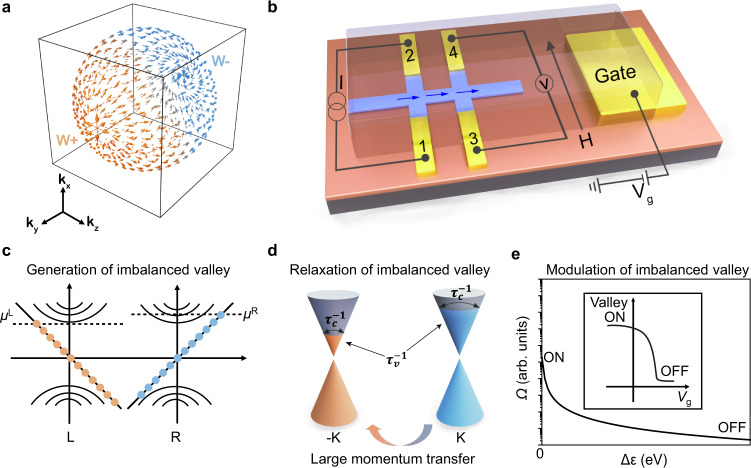
Room-temperature valley transistors based on Weyl materials. **a** Non-trivial Berry curvature in the Weyl materials. The vector plots of the Berry curvature connect Weyl points (W + and W-) in the momentum space. **b** Schematic illustration of the device structure of valley transistor with Weyl semiconductor. In this non-local structure, we apply constant current (*I*) through terminal 1–2 and detect the valley diffusion (blue arrow) through terminal 3–4 voltage (*V*). Electrostatic gating voltage (*V*_g_) is used for controlling the Fermi level (*E*_F_) and Berry curvature. **H** is the applied magnetic field. **c** Generation of imbalanced valley based on the chiral anomaly. L and R are left-and right-handed Weyl Fermions, respectively. *μ*^L^ and *μ*^R^ are the chemical potentials of left and right chirality, respectively. **d** Relaxation of imbalanced valley through intervalley scattering. The valley pseudospin (indicated by node position ±K) imbalance relaxes through intervalley scattering at a rate of ${\tau }_{v}^{-1}$ since intravalley scattering (${\tau }_{c}^{-1}$) cannot change the chirality. **e** Modulating valley through controlling the strength of Berry curvature (Ω). Δε is the energy separation between *E*_F_ and Weyl point. The inset shows that electrostatic gating can shift *E*_F_ and Berry curvature to achieve “ON” and “OFF” state in the transfer curve.

The topological properties in Weyl materials arise from the non-trivial Berry curvature. The arrows in Fig. 1a show the flux of the Berry curvature from one Weyl point (W+, light brown) to the other (W-, blue). Non-local structure (Fig. 1b) can significantly reduce the effects of conventional Ohmic transport, which enables generating, propagating (the blue arrow), detecting and modulating valley transport. Based on the non-trivial Berry curvature, chiral anomaly generates the imbalanced valley polarization under parallel magnetic field and electric field (**B//E**)^19,20^ (Fig. 1c). Different from widely investigated excitonic valley polarization, the absence of strong Coulomb interaction can retain a long lifetime of imbalanced valley generated by chiral anomaly. The intervalley scattering determines the lifetime of valley during propagating (described by ${\tau }_{v}$), which requires a large momentum transfer (Fig. 1d)^12,21^. Theoretically, valley can reach a long transport length of 100 μm (${L}_{v}=\sqrt{D{\tau }_{v}}$, ${L}_{v}$ is the intervalley scattering length and $D$ is the charge diffusion coefficient)^12^. We can control valley transport by modulating Berry curvature, which determines the pumping rate ($w$) of imbalanced valley according to Eq. (1)^13,22^:

1 $$w=f\left(\varOmega \right)\frac{{e}^{3}}{4{\pi }^{2}{{{\hslash }}}^{2}}{{{{{\bf{E}}}}}}\cdot {{{{{\bf{B}}}}}}$$

where *e* is the electron charge, and $\hslash$ is the reduced Planck constant. As Berry curvature is highly sensitive to the energy separation (Δ*ε*) between *E*_F_ and Weyl point^23,24^, electrostatic gating can effectively control Berry curvature and valley transport. Figure 1e shows a typical distribution of the Berry curvature as a function of Δ*ε*^24^. When *E*_F_ shifts close to the Weyl point, Δ*ε* ≈ 0, the Berry curvature is strong, which can generate high valley resistance; while *E*_F_ moves away from the Weyl point, Δ*ε* » 0, the Berry curvature rapidly decays, showing low valley resistance. Therefore, we can modulate the valley resistance by controlling the position of *E*_F_ to switch valley FETs (inset of Fig. 1e).

Compared to the tested valley transport in topological semimetals^14,25^, the modulation of *E*_F_ and valley transport in Weyl semiconductor is more efficient due to the tunable carrier density (Supplementary Table 1). Te is a Weyl semiconductor that can exhibit exotic transport behaviours from the Weyl point and non-trivial Berry curvature^23,26^. The intrinsic defects of Te make its *E*_F_ near the valence band maximum^23,27^, which results in a small energy separation (Δε of about 20 meV) between the *E*_F_ and the Weyl point (Supplementary Fig. 1, Supplementary Fig. 2 and Supplementary Note 2) and facilitates electrostatic modulation of transistors based on Berry curvature (Supplementary Fig. 3).

**Fig. 2:**
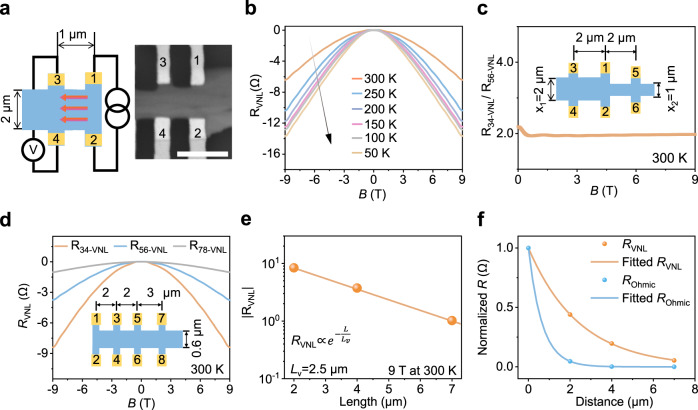
Temperature-, width- and length-dependent valley transport characteristics. **a** Schematic and scanning electron microscopy image of the “H” geometry Te valley device. After applying the constant current *I*_d_ through terminal 1–2, imbalanced valley (red arrow) propagates to the detection terminal 3–4. The scale bar is 3 μm. **b** Temperature-dependent valley transport from 50 to 300 K. Valley non-local resistance (*R*_*VNL*_) decreases as the temperature increases. **c** The ratio of non-local valley resistances under different widths. *R*_34-VNL_ and *R*_56-VNL_ are corresponding to the non-local valley resistance from terminals 3–4 and 5–6, respectively. Inset is the schematic of width-dependent valley device with width of x_1_ = 2 μm and x_2_ = 1 μm. **d** Length-dependent valley transport as a function of **B** at 300 K. Inset is the schematic of the length-dependent valley device with the channel of 2 μm (*R*_34-VNL_), 4 μm (*R*_56-VNL_) and 7 μm (*R*_78-VNL_). **e** Length-dependent valley transport under 9 T at 300 K. | *R*_VNL_ | is shown as a function of channel length in a semi-log curve. The yellow line is a visual guide that corresponds to a valley diffusion length (*L*v) of ~2.5 μm, fitted by Eq. (2). **f** Decay curves of the normalized valley and Ohmic resistance as a function of length. Valley transport can persist over much longer distances than charge transport.

Conventional Ohmic transport decays very fast due to unavoidable phonon scattering. In contrast, valley transport exhibits low-loss transport characteristics because of its robustness to low-energy phonon scattering^4,15^. The non-local device structure (Fig. 2a) allows the detection of the valley non-local resistance (*R*_*VNL*_)^25,28^. For the Weyl-based valleytronics, *R*_*VNL*_ follows the relationship in Eq. (2)^12,25^:

2 $${R}_{{{{{{\rm{VNL}}}}}}}\propto {e}^{-\frac{L}{{L}_{v}}}$$

where *L* is the length between the Hall bar terminals and *L*_*v*_ is the intervalley scattering length. By applying a constant current through terminals 1 and 2, we measure the local resistance (*R*_*L*_) between terminals 1 and 2 and the non-local resistance (*R*_*NL*_) between terminals 3 and 4. The tested *R*_*NL*_ includes a mixed contribution from Ohmic non-local resistance (*R*_*ONL*_) and *R*_*VNL*_. Since the non-local response from *R*_*VNL*_ vanishes at **B** = 0, we can extract *R*_*ONL*_ from *R*_*NL*_ (details in Supplementary Note 3). The temperature-dependent *R*_VNL_ (Fig. 2b) shows that the valley resistance decreases as the temperature increases from 50 to 300 K. At room temperature, we can still observe significant non-local valley resistance (*R*_VNL_ = −6.4 Ω under 9 T) because intervalley scattering requires a large momentum transfer.

The valley transport characteristics can be further supported by width-dependent and length-dependent non-local valley transport measurements. Supplementary Figure 4 presents *R*_*VNL*_ with different channel widths. *R*_*34-VNL*_ (with a width of 2 μm) and *R*_*56-VNL*_ (with a width of 1 μm) exhibit −2.1 and −1.1 Ω, respectively. The ratio of *R*_*34-VNL*_ to *R*_*56-VNL*_ is ~2 (Fig. 2c), matching well with the width ratio of 2 (inset of Fig. 2c). Supplementary Fig. 5 shows the reproducible temperature- and width-dependent valley transport results, supporting the valley transport behaviours in Te.

In addition, we investigate the length-dependent valley transport behaviour with different channel lengths of 2 μm, 4 μm and 7 μm. Figure 2d shows *R*_*VNL*_ as a function of the magnetic field, clearly exhibiting length-dependent valley transport characteristics. The valley resistance is still sufficiently detectable (*R*_*VNL*_ = −1.0 Ω under 9 T), even with a long transport distance of 7 μm. Figure 2e shows the |*R*_*VNL*_| as a function of channel length in a semi-log curve, fitting well with Eq. (2). The extracted *L*_v_ of 2.5 μm is much longer than the mean free path of electrons (tens of nm in Si at room temperature^29^), which reveals the low-loss transport characteristics in Te. Figure 2f presents the length dependence of the normalized valley and conventional charge transport contribution. The Ohmic resistance shows fast decay with the increase of distance (described by the Drude model). In contrast, the valley resistance can still be detected over a long distance because the relaxation process of valley transport involves a large quasi-momentum transfer.

We further investigate the carrier-density-dependent valley transport (Supplementary Fig. 6). We conducted the Hall tests of different samples (Supplementary Fig. 6a) and extracted the hole density values, ranging from 6.3 × 10^13^ cm^−2^ (grey line) to 9.2 × 10^13^ cm^−2^ (pink line) due to different concentrations of intrinsic Te vacancies^23^. For the Te sample with a higher carrier density, the device shows a higher valley resistance (Supplementary Fig. 6b). This trend arises from the carrier-density-dependent E_F_ and Δε. For the Te flakes with the higher hole density, it has a lower E_F_ position and smaller Δε, which results in a stronger Berry curvature and higher valley resistance. It is noteworthy that these topological properties are highly resistant to the structural defects^30^. We also simulate the voltage potential distribution of conventional charge transport in the non-local configuration under **B**//**E** (Supplementary Fig. 7). The simulation results suggest that the signal from conventional charge diffusion (Ohmic contribution) is negligible due to its short mean free path of electrons (e.g. tens of nm in Si), consistent with the previous non-local chiral anomaly work^13^. These length-, width- and carrier-density-dependent results reveal the presence of low-loss valley transport based on the chiral anomaly in the Te samples.

### Room-temperature volatile and non-volatile valley transistors

**Fig. 3:**
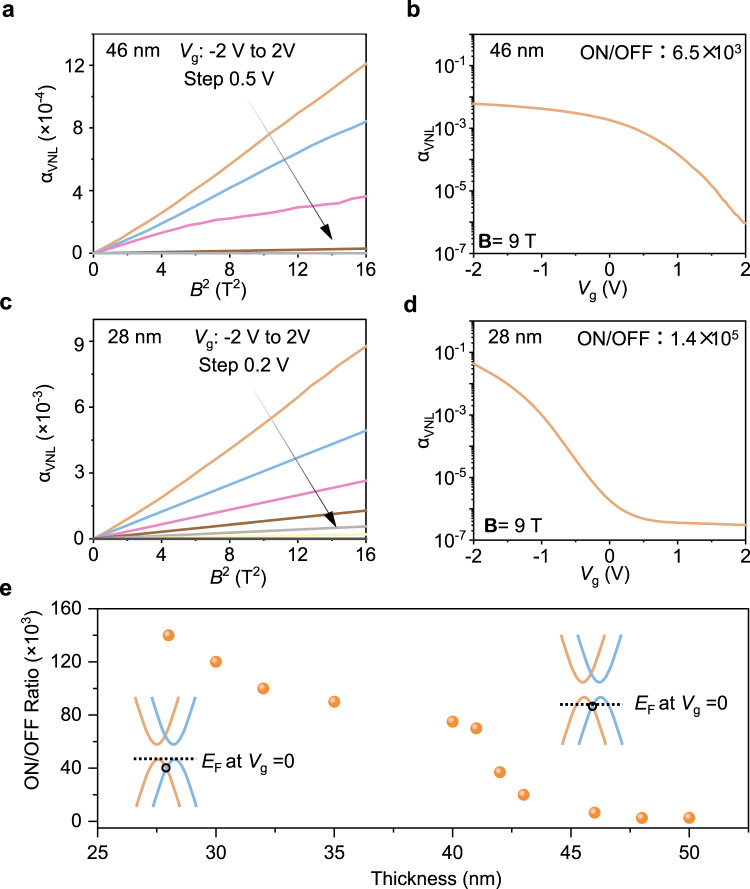
Volatile valley transistors at room temperature under B//E. **a** Output and **b** transfer curves for the 46 nm thick Te. α_VNL_ is a dimensionless coefficient, which is defined as |*R*_*VNL*_*/R*_*L*_ | . **c** Output and **d** transfer curves for the 28 nm thick Te. **e** ON/OFF ratio of valley FETs as a function of the sample thickness under **B** = 9 T.

To efficiently modulate the strength of Berry curvature and valley resistance at room temperature, we adopt ionic liquid (DEME-TFSI) with a high equivalent capacitance of ~10 μF/cm^2^ for electrostatic gating. Researchers use the dimensionless coefficient α_VNL_ = |*R*_*VNL*_*/R*_*L*_ | to quantitatively describe the relative strength of the valley resistance^12,25^. Figure 3a shows α_VNL_ as a function of ***B***^2^ of a 46 nm thick Te flake, exhibiting a linear shape under different gating voltages. The α_VNL_-*V*_g_ curve shows an ON/OFF ratio of 10^3^ (Fig. 3b). For this thick sample, *E*_F_ is close to the Weyl point, which results in strong valley transport at *V*_g_ = 0. For the 28 nm thick Te flake, it also exhibits nearly linear α_VNL_-*B*^2^ curves under the *V*_g_ from −2 to 2 V (Fig. 3c). The α_VNL_-*V*_g_ curve of the Te valley FET shows an ON/OFF ratio of 10^5^ (Fig. 3d), which is comparable to that of reported charge-based Te FETs (10^4^–10^6^) and much higher than that of other valley FETs (10^2^–10^3^) in the existing literature (Supplementary Table 2). The strong dependence of valley transport on the position of *E*_F_ in the Weyl semiconductor Te leads to a high ON/OFF ratio of valley FETs. When we apply negative gate voltages, negative ions [TFSI]^-^ are adsorbed on the Te flake, which gives rise to an ultrathin electrical double layer at the surface of Te. The induced holes downshift *E*_F_ close to the Weyl point of Te, increasing the valley resistance. When we remove the gate voltage, the valley resistance rapidly decays, exhibiting volatile behaviour and unique ion dynamics.

Figure 3e shows the thickness-dependent ON/OFF ratio of valley FETs. As the thickness of Te flakes increases from 28 to 50 nm, the ON/OFF ratio of Te valley devices under 9 T decreases from 1.4 × 10^5^ to 2.6 × 10^3^. To reveal the thickness-dependent carrier densities and *E*_F_, we carried out the Hall resistance experiments for the samples with different thicknesses (Supplementary Fig. 8a), along with the theoretical calculation (Supplementary Fig. 8b, c) and mechanism analysis (Supplementary Note 4). Consistent with existing works based on solution-prepared Te, higher hole carrier density exists in the thicker Te samples^26,31,32^. For performance comparison, we used the 28 nm thick Te flake as the channel of charge-based FETs with ionic liquid. The *I*_d_-*V*_d_ curve of the Te charge-based FET exhibits a linear shape under ***V***_ds_ of 0 to 1 V and an ON/OFF ratio of 240 (Supplementary Fig. 9). This tested ON/OFF ratio is comparable to the Te charge-based FET works with a similar thickness on the high-*k* dielectric layer^32^ (Supplementary Table 2), but much smaller than our valley FETs. Implementing the ferromagnetic material interface or using the alternating current can potentially trigger valley transport in the Weyl materials without using the magnetic field^33,34^.

**Fig. 4:**
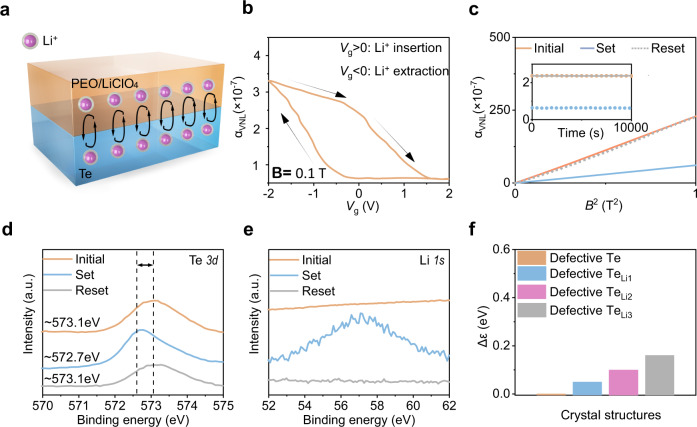
Non-volatile valley transistors based on reversible ion insertion/extraction at room temperature. **a** Schematic of non-volatile valley transistors with the solid-electrolyte PEO/LiClO_4_. The black arrow indicates the movement of Li ions. **b** Transfer curves with hysteresis under **B**//**E** and **B** = 0.1 T. The black arrow indicates the sweeping direction of *V*_g_. **c** Output curves of different states. Inset shows the non-volatile valley states under the retention tests of 10,000 s. Set corresponds to the gating of +2 V with 1 s, while reset is the gating of −2 V with 1 s. **d** XPS *3d* spectra of Te. The vertical dashed lines and the arrow indicate the shift of peaks. The peak shifts from ~573.1 eV (orange) in the initial Te to ~572.7 eV (blue) after +2 V pulse (set). With a further gating of −2 V pulse (reset), the peak moves back to ~573.1 eV (grey). **e** XPS Li *1* *s* spectra. The +2 V pulse results in the emergence of a rough peak (blue), while the following −2 V pulse causes this peak to disappear (grey). **f** Energy separation between *E*_F_ and the Weyl point for defective Te (1.23% Te vacancies), defective Te_Li1_ (1.23% Te vacancies and 1.23% Li doping), defective Te_Li2_ (1.23% Te vacancies and 2.46% Li doping) and defective Te_Li3_ (1.23% Te vacancies and 3.69% Li doping).

In addition to volatile behaviours of valley FETs, we adopt stable solid-state electrolyte PEO/LiClO_4_ to realize non-volatile valley states (Fig. 4a). Li^+^ can be inserted into Te flakes under positive gate voltages and be extracted under negative gate voltages^35,36^. The Li^+^ insertion can upshift the *E*_F_ and enlarge the Δε (Supplementary Fig. 10), which results in the non-volatile valley resistance. Ion doping is a common technique for investigating the physical properties of topological materials by only shifting the *E*_F_ without changing the band structure^37,38^. The Weyl point is topologically protected and robust against external disturbance (thermal, ion insertion, etc)^39,40^. Figure 4b presents the transfer curves in the sequence of 0 → +2 V → 0 → −2 V → 0. For ***V***_**g**_ > 0, Li^+^ is adsorbed and inserted into the Te flakes. Thus, the valley resistance decreases from 0 → +2 V and keeps stable during +2 V → 0. For *V*_g_ < 0, due to the coexistence of Li^+^ extraction and the electrical double layer, the valley resistance increases during 0 → −2 V; as the ***V***_**g**_ varies from −2 V → 0, the strength of the electrical double layer becomes weak, decreasing the valley resistance.

Figure 4c shows the output curves of different states before and after gating. After applying *V*_g_ = +2 V for 1 s (set operation), the valley resistance decreases due to the non-volatile Li^+^ insertion and larger Δ*ε*. Then, we apply *V*_g_ = −2 V for 1 s to reset the devices, which results in the valley resistance close to the initial state, indicating the reversible ion insertion/extraction. We further investigated the stability of non-volatile states through the retention tests of 10,000 s (inset of Fig. 4c). The tested curves show slight variation during the retention tests, revealing the stable non-volatile valley states based on valley transport.

To validate the working mechanism of Li^+^ insertion/extraction, we carry out X-ray photoelectron spectroscopy (XPS) characterization of the Te samples at different stages: initial Te and Te with *V*_g_ = ±2 V. Upon applying *V*_g_ = +2 V for 1 s, the XPS spectra of the Te sample (Fig. 4d, blue curve) shows a peak at ~572.7 eV between the peak of Te^0^ (~573.1 eV) and the peak of Li_2_Te (572.4 eV)^41^. This characteristic peak is consistent with the Li spectra (Fig. 4e), indicating that Li^+^ exist in the Te after applying *V*_g_ = +2 V for 1 s. Following *V*_g_ = −2 V for 1 s, the peak of Te (Fig. 4d, grey curve) is similar to that of the pristine Te, and the Li peak disappears (Fig. 4d, grey curve), indicating the extraction of Li^+^. In addition, the XPS spectra (Supplementary Fig. 11) show the absence of Cl peaks in the Te samples at different stages, suggesting the complete removal of PEO/LiCl_4_ after washing. These results reveal that Li^+^ can be successfully inserted and extracted by applying appropriate gate pulses.

Furthermore, we calculate the effects of carrier concentrations of ions on the non-volatile shift of *Δε* through the density functional theory (Fig. 4f and Supplementary Fig. 12). In the Li-inserted defective Te_Li1_ structure (1.23% Li intercalation), Δ*ε* increases to 0.05 eV. With more Li inserted into the Te_Li2_ structure (2.46% Li concentration), Δε is 0.10 eV. As the Li concentration increases from 2.46 to 3.69%, *Δε* further increases by 0.16 eV. These results reveal that Li insertion can be used to enlarge the Δ*ε* of Te step-by-step with increasing Li concentration. Li insertion/extraction can quantitatively control multiple non-volatile valley resistance states.

### Synaptic valley transistors for low-power neuromorphic computing

Conventional charge-based synaptic transistors suffer from relatively large readout power (nW to μW) in the inference process for neuromorphic computing^16–18,42–44^. It is of significance to adopt a low-loss transport mechanism to realize low-power computing^45^. Synaptic valley transistors show the potentials of achieving a high signal-to-noise ratio with low readout power due to the robust valley transport.

**Fig. 5:**
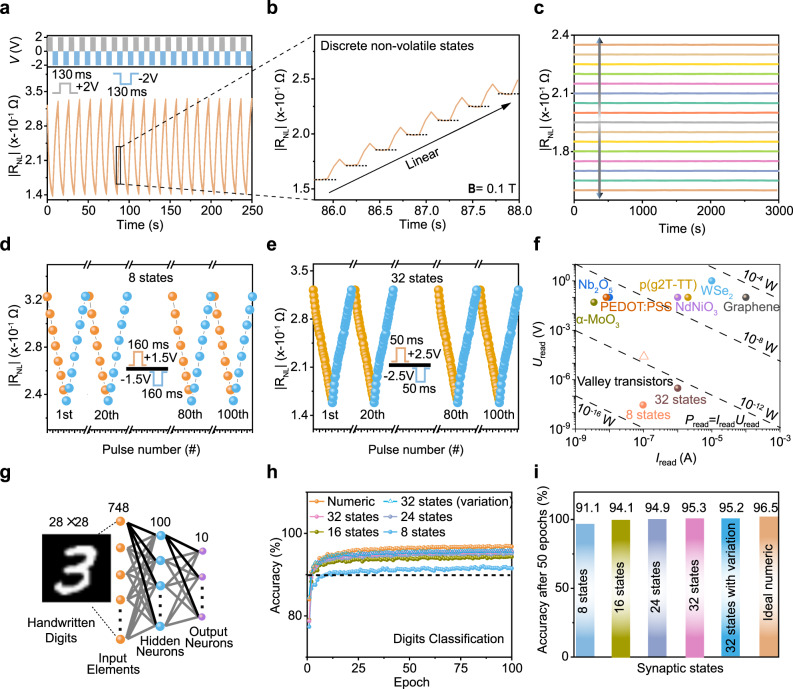
Synaptic valley transistors with linear and long-term memory characteristics for neuromorphic computing. **a** Potentiation and depression states under the control of voltage pulses with the solid-state electrolyte PEO/LiClO_4_. *V*_g_ = +2 V is applied to adsorb and intercalate Li^+^ into Te and move *E*_F_ away from the Weyl point. *V*_g_ = −2 V is applied to relax and extract Li^+^ and move *E*_F_ back. The test temperature is 300 K under **B** = 0.1 T. **b** A zoomed-in view of **a** with multiple linear states. The dash lines indicate the stable states. **c** Stable valley states under retention tests of 3000 s. *V*_g_ > 0 for reducing the valley resistance and *V*_g_ < 0 for increasing the valley resistance. **d**, **e** Cycle-to-cycle performance of valley synapses with **d** 8 and **e** 32 stable states under the applied constant current of 100 nA and 1 μA, respectively. **f** Readout current and voltage in our valley transistors and reported works. The dash lines are corresponding from 10^−16^ to 10^−8^ W. **g** Artificial neural network for classifying handwritten digits. The linear and stable valley states are used for the connected synapses between neurons. Inset shows a three-layer artificial neural network for classifying handwritten digits in the MNIST database. **h** Neuromorphic classification based on the non-volatile valley transistors. The black dash line corresponds to the 90% recognition accuracy. **i** Classification accuracy after 50 epochs for different non-volatile states and ideal numeric.

Training of the ANN requires programmable non-volatile states and weight updating. Through ion insertion/extraction into Te channel with the electrolyte PEO/LiClO_4_, we can realize non-volatile resistance modulation for emulating synaptic functions (Fig. 5). By applying writing voltage pulses (+2 V/−2 V, 130 ms) to the gate terminal of the valley FETs, the device shows depression/potentiation of the valley resistance with 20 distinct states (Fig. 5a), which correspond to long-term depression (LTD) and long-term potentiation (LTP) synaptic behaviours. By applying a gate voltage pulse (***V***_g_ = −2 V, 130 ms), Li^+^ insertion and narrower Δ*ε* cause an increase in the valley resistance. After the removal of the gate voltage (Fig. 5b), the valley resistance first drops, and then remains stable at a relatively higher level compared to the pristine one because of Li^+^ extraction.

We demonstrate LTP and LTD behaviours with 8, 16, 24 and 32 stable valley resistance states under different pulse configurations (Supplementary Fig. 13) under **B** = 0.1 T. As summarized in Supplementary Fig. 14, our devices show linear weight update behaviour with high symmetry (e.g. the nonlinearity of 0.012/−0.442 and asymmetry of 0.454 for 8 states). The multiple resistance states show negligible deviation for 3000 s (Fig. 5c), indicating good retention of non-volatile valley FET states. Thus, the relatively low magnetic field (**B** = 0.1 T) can trigger the valley transport and retain linear, analogue, non-volatile valley synaptic weights for neuromorphic computing. The cycling tests (Fig. 5d, e and Supplementary Fig. 15) exhibit low cycle-to-cycle variation for 8 to 32 states (e.g. 0.371% variation for 32 states). The LTP and LTD characteristics exhibit highly linear features, paving the way for the realization of ANN with high learning accuracy.

The valley transport is robust against thermal perturbation^25^, which enables to use the relatively low readout current (100 nA level) and voltage (10 nV level) for readout while maintaining a high signal-to-noise ratio. Figure 5f compares readout voltage (*U*_read_), readout current (*I*_read_) and readout power (*P*_read_) with reported works (details in Supplementary Table 3). The readout power can be calculated from *P*_read_ = *U*_read_*I*_read_^16^. The valley devices show 100 fW level (solid brown ball) for 32 states under the read current of 1 μA, and ~ fW level (solid orange ball) for 8 states under the read current of 100 nA (sufficient to ensure a relatively high signal-to-noise ratio and linear synaptic behaviours). The readout process of synaptic valley transistors requires Ohmic-based drive current through the local terminal, which gives rise to the power consumption of ~pW level (hollow pink triangle in Fig. 5f, details in Supplementary Note 5). In this work, the synaptic valley transistor exhibits low power consumption during readout (Ohmic contribution of ~pW and valley contribution of ~fW), orders of magnitude lower than conventional charge-based synaptic devices with the readout power of nW to μW levels in the inference process^16,17,43,46–50^. The valley transport is robust against low-energy events in the environment, which allows us to observe discrete and stable synaptic states with ultralow reading current/voltage and provides the potential for realizing low-power neuromorphic computing.

We further simulate a three-layer ANN based on valley synaptic behaviour to classify handwritten data with a widely used Modified National Institute of Standards and Technology (MNIST) database. The detailed training procedures for weight update based on valley transistors (Fig. 5g) are summarized in the Method and Supplementary Note 6. As the number of states increases from 8 to 32, the classification accuracy gradually increases (Fig. 5h). With 8 states, the recognition accuracy is higher than 91% due to the ultralow nonlinearity (0.012/−0.442); with 32 states, the accuracy reaches 95.2%. Even with the consideration of cycle-to-cycle variation (0.371%), the classification accuracy is still higher than 95%, which is close to the accuracy value for the ideal numeric simulation (96.5%). The classification accuracy after 50 training epochs (Fig. 5i) is comparable to the state-of-the-art performance of neuromorphic computing works^43,44^. We demonstrate low-power neuromorphic computing based on valley synaptic devices by utilizing robust valley transport.

## Discussion

In summary, we demonstrate room-temperature valley transistors with a Weyl semiconductor. Our devices show robust valley transport characteristics, which can propagate over 7 μm at room temperature. Through the electrical generation and modulation of valley transport, the valley transistors show 10^5^ ON/OFF ratio. We also demonstrate the non-volatile change of valley resistance through ion insertion/extraction by modulating non-trivial Berry curvature, which enables the emulation of the synaptic functions with 32 linear, high symmetry and discrete non-volatile states for ANN. Non-volatile valley FET exhibits low readout power (Ohmic contribution of ~pW and valley contribution of ~fW), a few orders of magnitude lower than reported synaptic works (from nW to μW). The accuracy reaches up to 95.2% for neuromorphic classifying handwriting data. With the low-loss characteristics of the valley degree of freedom, our valley transistor provides an alternative to conventional charge-based devices for low-power neuromorphic computing.

## Methods

### Device fabrication

We prepared Te flakes by hydrothermal methods^32^ and transferred them to a Si substrate with 300 nm thick SiO_2_. The thickness of Te flakes is more than 10 nm, which can retain the band structure similar to bulk Te and exhibit magnetotransport signatures of the Weyl point^51^. Electron beam lithography was used to define the pattern of metal electrodes in a Hall bar configuration with a side gate. Metal contacts were prepared by thermal evaporation of Au (80 nm) at a rate of 0.3 Å/s under a vacuum of 3 × 10^−7^ Torr. The Te flake for the non-local valley transport measurement was etched by a focused ion beam to achieve the “H” configuration^25^. A low beam current of 10 pA at 20 kV was adopted in the etching process to avoid severe damage to the sample.

### Device characterization

Electrical measurements were carried out with the Physical Property Measurement System from Quantum Design (DC resistivity and ETO modules with the typical noise floor of 1 nV/rtHz) and the Keithley 4200. After applying a constant current (*I*_constant_), the equipment directly tested the voltage signal (*U*), and exported the resistance signal (*R*). To modulate the valley FET, a droplet of the ionic liquid DEME-TFSI was used to cover the Te flake and the side gate electrode. Before the test, the device was kept under a high vacuum of 4 × 10^−8^ Torr for 24 h to remove the water. We also chose solid-state PEO/LiClO_4_ as the electrolyte because it is stable and can act as a source of Li ions. PEO/LiClO_4_ (PEO and LiClO_4_ in methanol with a mass ratio of 9:1) was coated on the sample, and then the sample was heated at 70 °C for 30 min to remove the solvent before the electrical tests. We characterized non-volatile valley synaptic behaviours at the temperature of 300 K and the magnetic field of 0.1 T. We extracted the carrier density based on the linear part of the Hall resistance curves (e.g. −4T ≤ **B** ≤ 4 T region in Supplementary Fig.  1).

### Material characterization

Scanning electron microscopy images were acquired by a JEOL Model JSM-6490. XPS spectra were acquired by a Thermo Fisher Nexsa. For the gated Te, the samples were carefully washed with water and methanol to remove PEO/LiClO_4_ before XPS characterization.

### First-principles calculations

The band structure and density of states calculations were carried out using density functional theory (DFT) implemented in the Vienna Ab initio Simulation Package (VASP)^52^. The Perdew-Burke-Ernzerhof-type generalized gradient approximation^53^ and the projector augmented-wave (PAW) method were employed^54^. A plane-wave basis set with a default energy cut-off and a 16 × 16 × 12 k-point mesh was used. A 3 × 3 × 3 Te supercell was used to calculate the density of states with or without Te vacancies. After removing one Te atom (marked by the yellow ball, Supplementary Fig. 2b) from the supercell, the vacancy density in the calculated deficient Te sample was 1/81 (~1.23%).

### Modelling the contribution of conventional charge transport

We modelled the distribution of non-local voltages from Ohmic contribution in the COMSOL Multiphysics. As conventional charge transport can be described by the Drude model, the contribution of charge when **B**//**E** (*σ*_||_) can be described by the Equation (3)^13^:

$${\sigma }_{\parallel }={\sigma }_{0}\left(\begin{array}{ccc}1 & 0 & 0\\ 0 & \frac{1}{1+{(\mu {{{{{\bf{B}}}}}})}^{2}} & \frac{\mu {{{{{\bf{B}}}}}}}{1+{(\mu {{{{{\bf{B}}}}}})}^{2}}\\ 0 & -\frac{\mu {{{{{\bf{B}}}}}}}{1+{(\mu {{{{{\bf{B}}}}}})}^{2}} & \frac{1}{1+{(\mu {{{{{\bf{B}}}}}})}^{2}}\end{array}\right)$$

where *σ*_0_ = *n*e*μ*, *n* is the carrier density, e is the electron charge, *μ* is the mobility.

### Neuromorphic computing simulations

A three-layer ANN was used to execute supervised learning over the training examples. There are 748 input elements, 100 hidden neurons and 10 output neurons. The experimental non-volatile valley resistance states are the synaptic weights between connected neurons. The MNIST dataset, a large image version (28 × 28 pixels) of handwritten digits, was used for training and classification. The recognition accuracy was compared with the test examples in a single training epoch.

## Supplementary information

Supplementary information

## Data Availability

Relevant data supporting the key findings of this study are available within the article and the Supplementary Information file. All raw data generated during the current study are available from the corresponding authors upon request.

## Code availability

The codes for the simulations are available from the corresponding author upon request.
