# Supplementary material for: Room-temperature valley transistors for low-power neuromorphic computing
Source: Nat Commun. 2022 Dec 15;13:7758. doi: 10.1038/s41467-022-35396-x (PMC9755139; doi:10.1038/s41467-022-35396-x)
Supplement: Supplementary file 1 — Supplementary information [file 41467_2022_35396_MOESM1_ESM.docx]

**Room-temperature** **valley transistors for low-power neuromorphic computing**

Jiewei Chen^1,2^, Yue Zhou^1,3^, Jianmin Yan^1,2^, Jidong Liu^4^, Lin Xu^1,2^, Jingli Wang^5^, Tianqing Wan^1^, Yuhui He^3^, Wenjing Zhang^4^, and Yang Chai^1,2^*

^1^ Department of Applied Physics, The Hong Kong Polytechnic University, Hong Kong, China

^2^ The Hong Kong Polytechnic University Shenzhen Research Institute, Shenzhen, China

^3^ Wuhan National Laboratory for Optoelectronics, Huazhong University of Science and Technology, Wuhan 430074, China

^4^ International Collaborative Laboratory of 2D Materials for Optoelectronics Science and Technology, Shenzhen University, Shenzhen 518060, China

^5^ Frontier Institute of Chip and System, Fudan University, Shanghai, China

*Corresponding author. Email: ychai@polyu.edu.hk

**This supplementary information includes:**

**Supplementary Figure 1 to 15**

**Supplementary Table 1 to 3**

**Supplementary Note 1 to 6**

**Supplementary References**


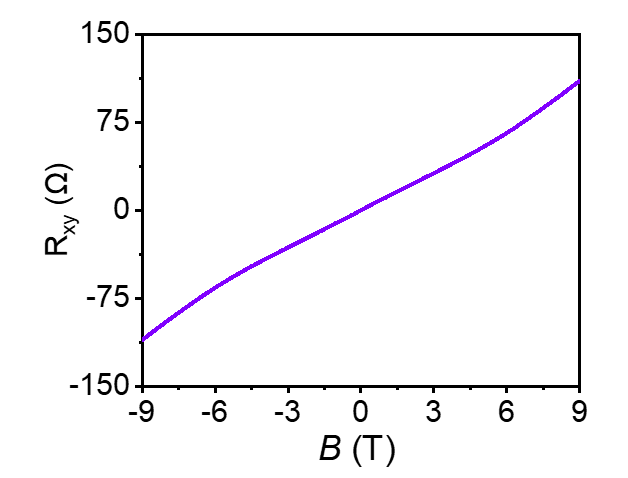


**Supplementary Figure 1 | Hall resistance of the Te sample at 300 K**. The high hole density 1.13×10^19^ cm^-3^ (*n*_2D_ =5.1×10^13^ cm^-2^, 45 nm) results from self-deficiency doping.

**
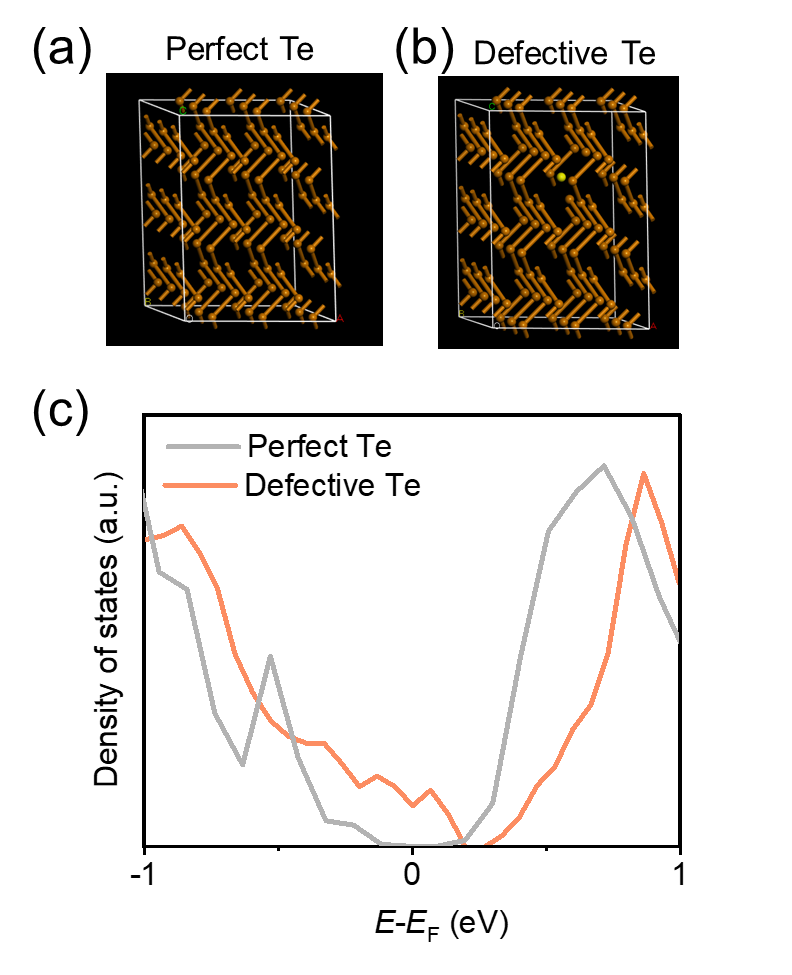
**

**Supplementary Figure 2| Calculation configuration and density of states for different Te structures.** (a) Configuration of perfect Te. (b) Configuration of defective Te (1.23% Te vacancies). The Te vacancy is marked as a yellow ball. (c) Densities of states of perfect and defective Te. The Fermi level of defective Te is near the Weyl point.

**
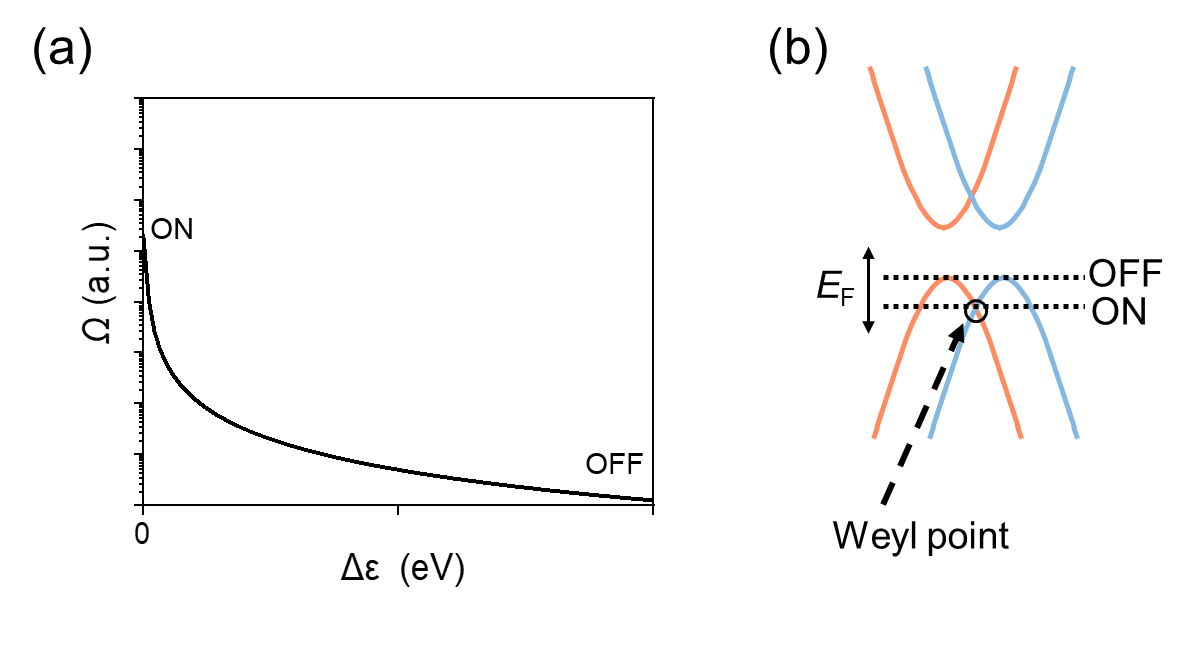
**

**Supplementary Figure 3 | Working mechanism of valley FET based on Weyl semiconductor Te.** (a) Berry curvature strength in Te as a function of the energy difference between the Weyl point and *E*_F_ by referring to the previous calculation results[^1^](#_ENREF_1)^,^[^2^](#_ENREF_2). (b) Modulation of *E*_F_ and Berry curvature to realize the "ON" and "OFF" states of valley transistors. The position of *E*_F_ is close to/far away from the Weyl point under the "ON" and "OFF" states, respectively.

**
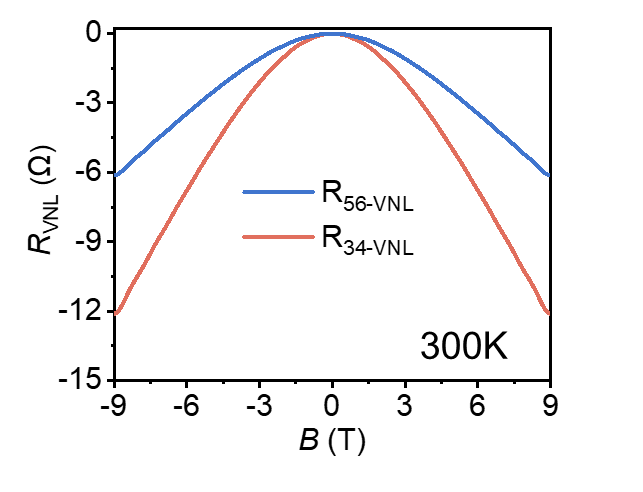
**

**Supplementary Figure 4 |** Width-dependent valley transport as a function of ***B*** at 300 K.


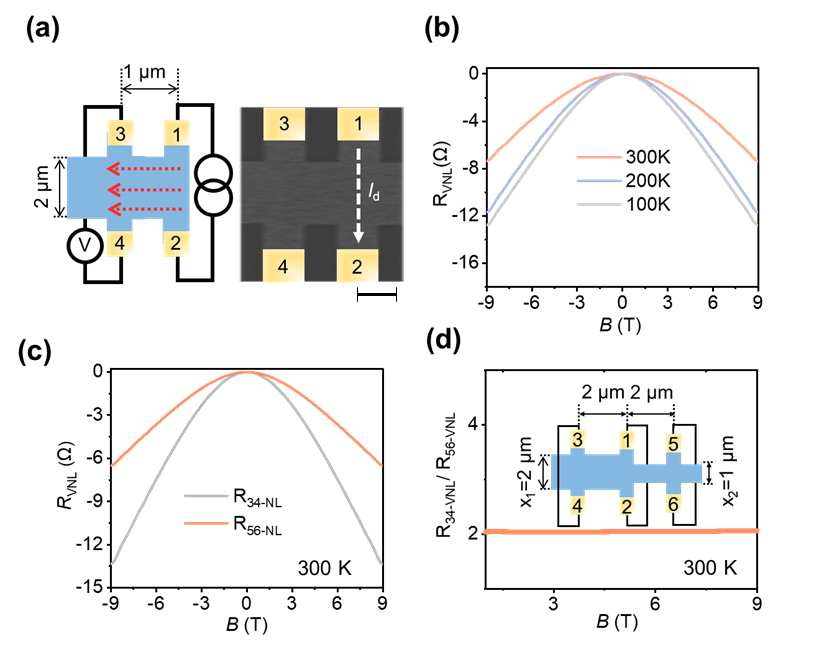


**Supplementary Figure 5 | Reproducible temperature- and width-dependent valley transport results based on Te.** (a) Schematic and scanning electron image of the etched nonlocal "H" structure. Terminal 1-2 for applying the constant current and terminals 3-4 for detecting the nonlocal voltage. The scale bar is 1 μm. (b) The temperature-dependent transport under different magnetic fields. (c) The width-dependent transport under different magnetic fields. R_34-VNL_ corresponds to the width of 2 μm, while R_56-VNL_ corresponds to the width of 1μm. (d) The ratio of R_34-VNL_ and R_56-VNL_. The inset shows the width-dependent testing configuration. Terminal 1-2 for applying the constant current. Terminals 3-4 and 5-6 for detecting the nonlocal voltage.

**
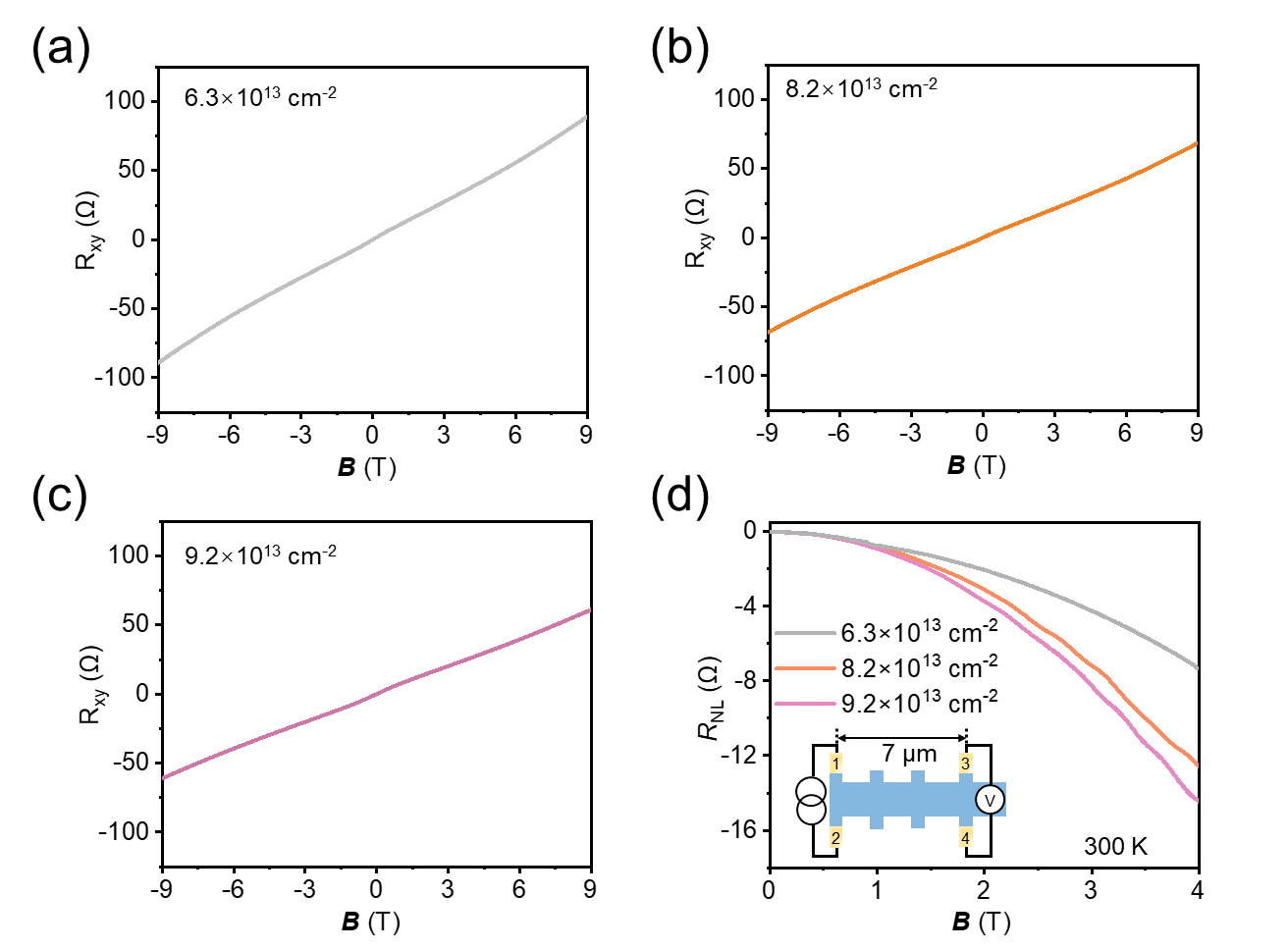
**

**Supplementary Figure 6 |** Carrier-dependent valley transport. (a-c) Hall resistance of Te samples with different carrier densities. (d) Carrier-dependent valley transport as a function of ***B*** at 300 K. Electrode pairs 1-2 for applying the constant current, while electrode pair 3-4 for detecting the voltage. As the Ohmic contribution is negligible in this electrode pair 3-4, we can observe *V*_VNL_≈*V*_NL_ (corresponding to *R_VNL_* ≈*R_NL_*) during the tests.

**
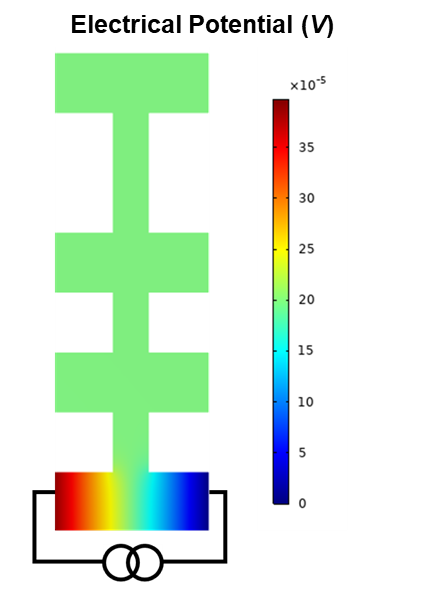
**

**Supplementary Figure 7 | Simulation of electrical potential distribution of conventional charge transport.** Nonlocal Te device under ***B***//***E*** at 300 K. Constant current is applied with 1 μA. ***B*** is set at 1T.


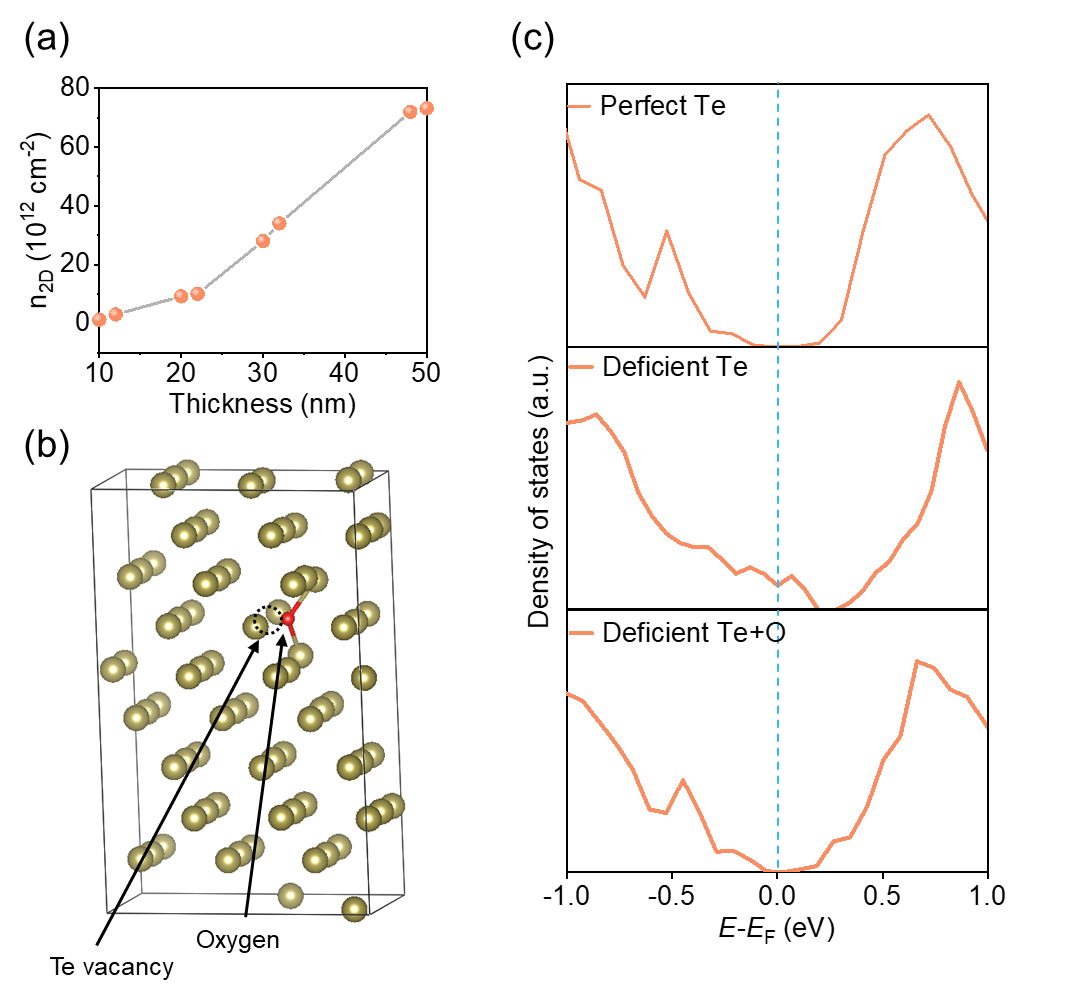


**Supplementary Figure 8 | Thickness-dependent carrier densities and related oxygen-passivated mechanism.** (a) Thickness-dependent Hall carrier densities for samples from 10 to 50 nm. (b) Atomic structures for deficient Te with bonded O. (c) Density of states for perfect Te, deficient Te and deficient Te with bonded O. ***E*_F_** lies in the bandgap for the perfect Te. For the deficient Te, ***E*_F_** is in the valence band and near the Weyl point. After oxygen passivation, ***E*_F_** is upshifted to the VBM, away from the Weyl point.

**
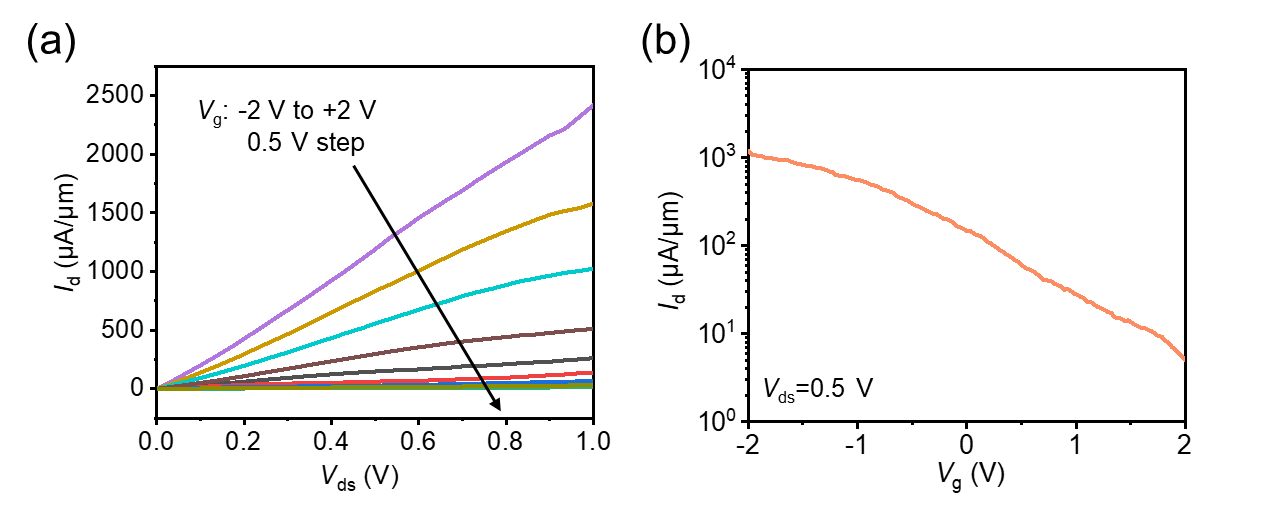
**

Supplementary Figure 9 | Charge-based FET in the 28-nm-thick Te flake as the valley FET. (a) Output curve *I_d_*-*V*_d_ in the charge-based FET as *V*_g_ increases from -2 V to 2 V with a step of 0.5 V. (b) Transfer curve in the Te Charge-based FET.


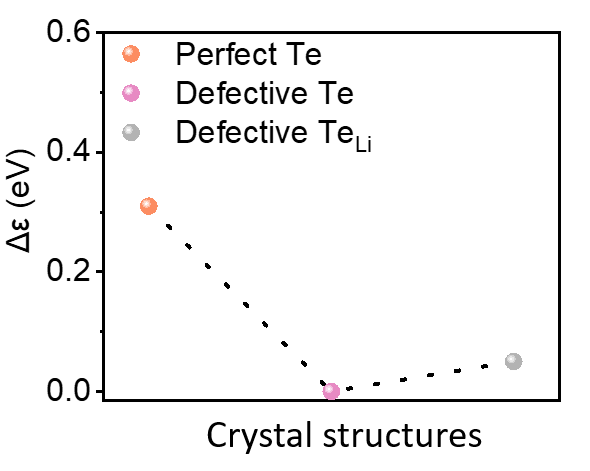


**Supplementary Figure 10 |** Energy separation between *E*_F_ and the Weyl point for perfect Te, defective Te (1.23% Te vacancies) and defective Te_Li1_ (1.23% Te vacancies and 1.23% Li doping).


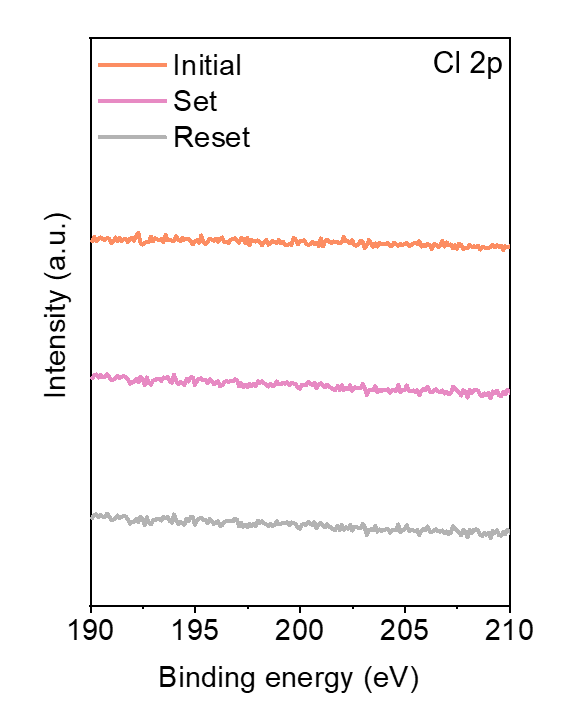


**Supplementary Figure 11 | XPS spectra Cl 2p spectra of Te under different situations.** There is no pronounced peak in the spectra, indicating the successful removal of PEO/LiClO_4_ after the washing.


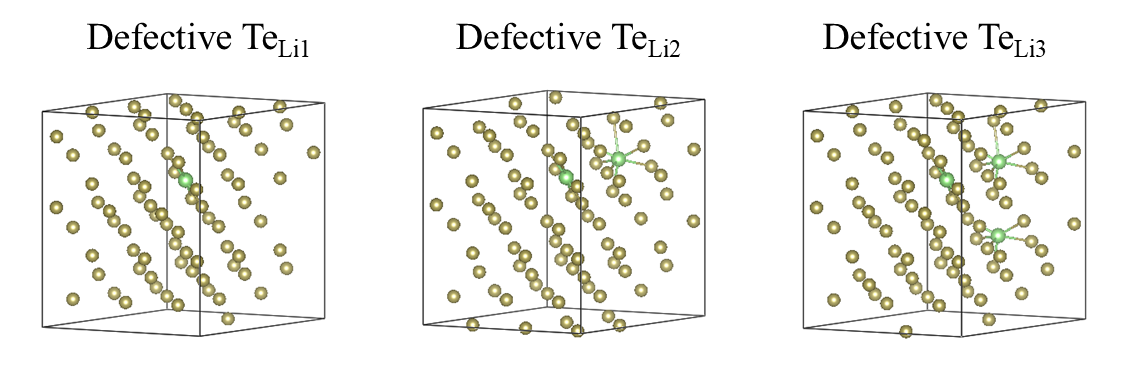


**Supplementary Figure 12 | Calculation configurations of defective Te with different Li doping concentration.** (a) Defective Te_Li1_ (1.23% Te vacancies and 1.23% Li doping). (b) Defective Te_Li2_ (1.23% Te vacancies and 2.46% Li doping). (c) Defective Te_Li3_ (1.23% Te vacancies and 3.69% Li doping). Doping Li is marked as the green ball.


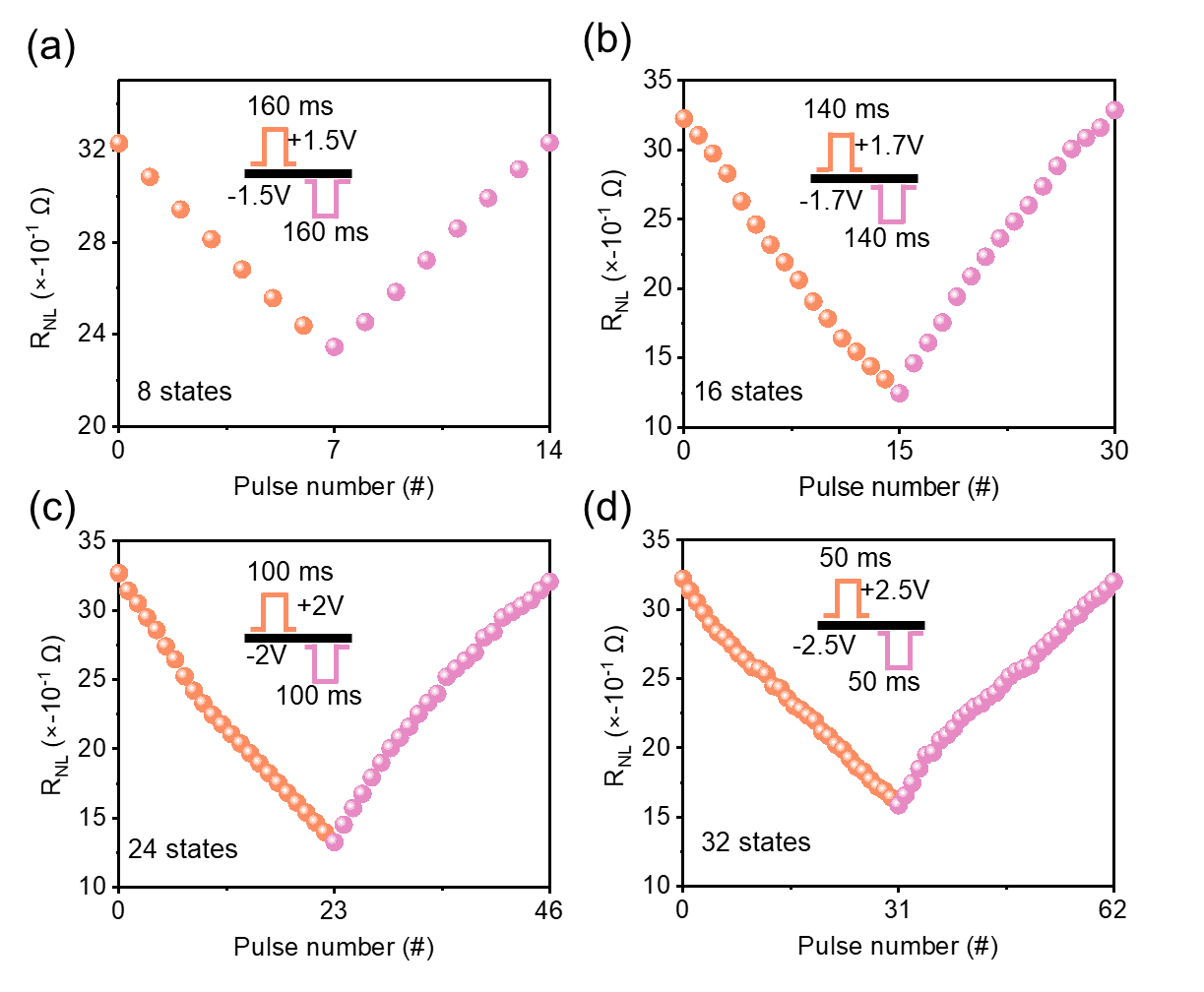


**Supplementary Figure 13 | Valley synapses with different stable states.** (a) 8, (b) 16, (c) 24 and (d) 32 discrete and linear states through electrochemical modulation.


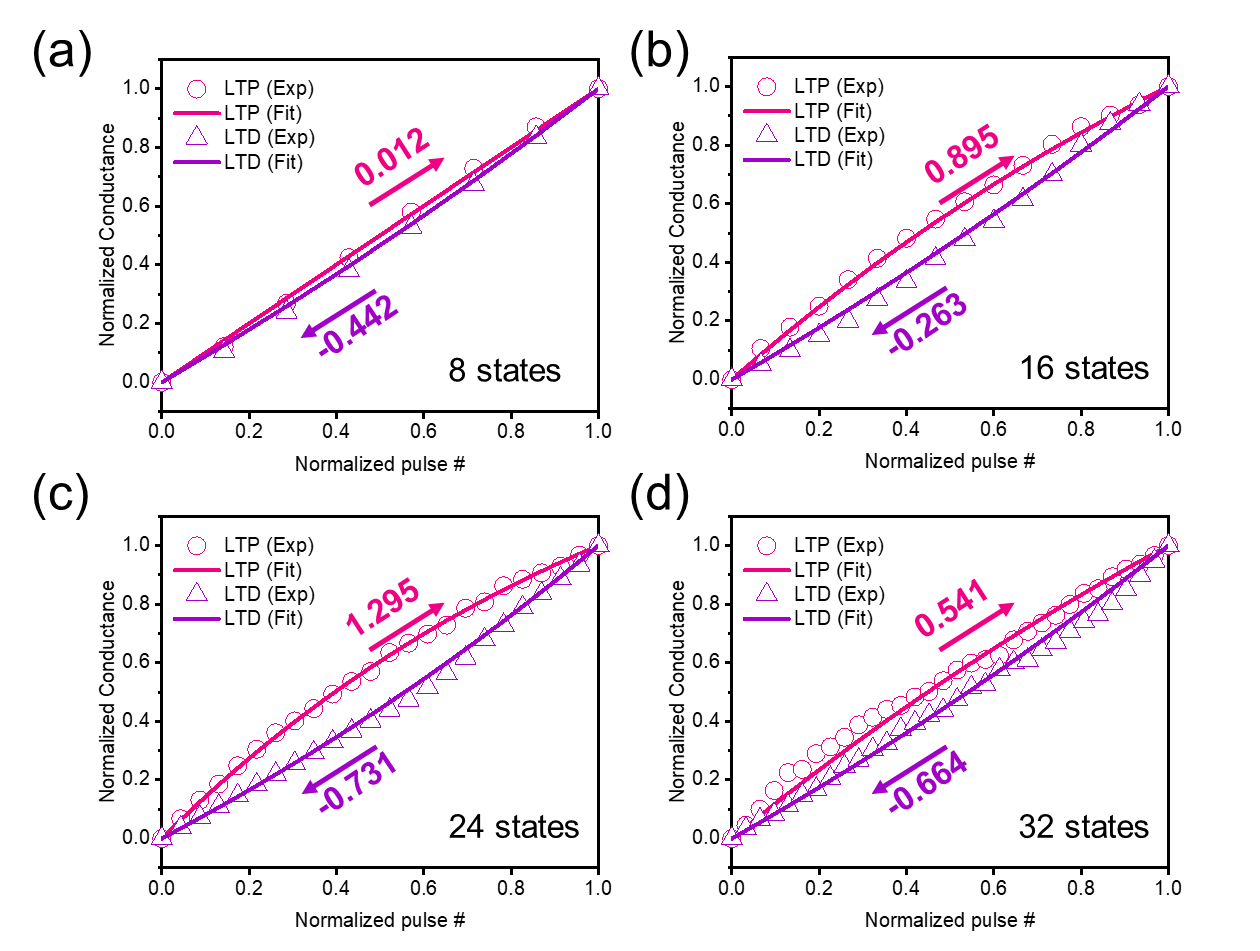


**Supplementary Figure 14 | Different nonlinearities of the long-term potentiation (LTP) and the long-term depression (LTD) to fit the experimental data.** All the data are normalized from 0 to 1 before the fitting. (a) The nonlinearities of LTP and LTD for 8 states are 0.012 and -0.442, respectively. (b) The nonlinearities of LTP and LTD for 16 states are 0.895 and -0.263, respectively. (c) The nonlinearities of LTP and LTD for 24 states are 1.295 and -0.731, respectively. (d) The nonlinearities of LTP and LTD for 32 states are 0.541 and -0.664, respectively.


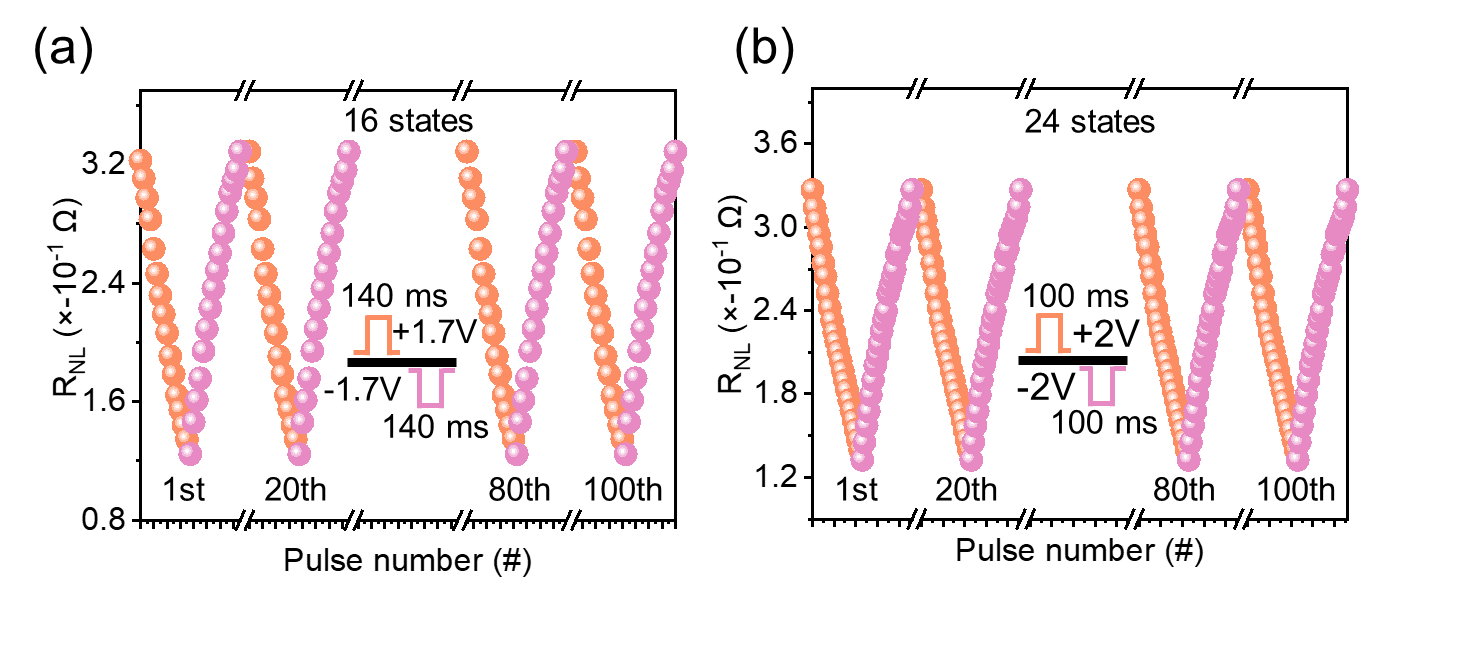


**Supplementary Figure 15 | Cycle-to-cycle performance of valley synapses with different stable states.** (a)16 and (b)24 discrete states under different stimulation pulses.

**Supplementary Table 1| Comparison of the topological semimetal and semiconductor for valleytronics.**

|  | Topological semimetal | Topological semiconductor |
| --- | --- | --- |
| Typical scheme of *E*_F_ in the band structure |  | 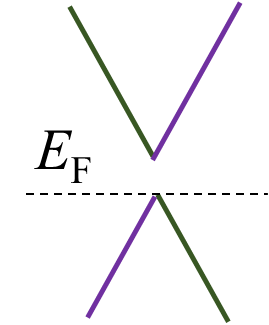 |
| Density of states around Weyl point | Strong | Relatively weak |
| Background influence | Strong | Relatively weak |
| Tunability of Fermi level and Berry curvature | Hard | Relatively easy |

**Supplementary Table 2| Performance comparison among our valley transistor, Te charge transistor and valley transistor at room temperature.**

| Works | Degree of freedom | ON/OFF ratio |
| --- | --- | --- |
| *Nat. Electron* 1, 228–236, 2018 | Charge | ~20 (30-nm thickness)  ~200 (25-nm thickness)  10^4^-10^5^ (10-nm thickness) |
| *Nat. Nanotechnol.* 15, 53–58, 2020 | Charge | ~10^4^ |
| *Nat. Nanotechnol* 15, 743-749, 2020 | Valley | 10^2^-10^3^ |
| **Our work** | **Valley** | **10^5^ (28-nm thickness)** |

**Supplementary Table III| Comparison of this work and reported charge-based synaptic works (**≥**8 nonvolatile states) for neuromorphic computing.**

| Works | Nonvolatile states | Nonlinearity | Asymmetry | $\frac{\mathrm{Weight}_{max}}{\mathrm{Weight}_{min}}$ | **Readout Power** | Accuracy of recognizing MNIST |
| --- | --- | --- | --- | --- | --- | --- |
| NdNiO_3_[^3^](#_ENREF_3) | ~9 | \ | \ | 170% | **100 nW** | 82% |
| WSe_2_[^4^](#_ENREF_4) | 64 | 0.56/-1.23 | 1.79 | 3000% | **10 μW** | \ |
| p(g2T-TT)[^5^](#_ENREF_5) | 100 | \ | \ | 200% | **200 nW** | \ |
| Graphene[^6^](#_ENREF_6) | 52 | 1.3/-1.1 | 2.4 | 300% | **10 μW** | 86% |
| PEDOT:PSS[^7^](#_ENREF_7) | 50 | \ | \ | 200% | **1 nW** | 97% |
| Nb_2_O_5_[^8^](#_ENREF_8) | 64 | 0.68/1.65 | 2.33 | 300% | **1 nW** | \ |
| α-MoO_3_[^9^](#_ENREF_9) | 50 | 0.31/0.31 | 0.62 | 160% | **~150 pW** | 87.3% |
| **This work** | 8  32 | 0.012/-0.442  0.541/-0.664 | 0.454  1.205 | 146%  210% | **~ fW (valley)**  **~100 fW (valley)** | 91%  95% |

**Supplementary Note 1. The generation and modulation of imbalanced valley based on chiral anomaly compared with other mechanisms**

Valley (a quantum degree of freedom) can act as an information carrier with high energy efficiency[^10-13^](#_ENREF_10). In the past 10 years, there have been intensive investigations on the intriguing physical properties of valley, mainly based on the photo- or electric-generation of valley polarization[^10^](#_ENREF_10)^,^[^14^](#_ENREF_14)^,^[^15^](#_ENREF_15).

Conventional photo-generation of valley polarization involves excitonic states, which suffer from the low working temperature, short exciton lifetime (~ps), and small transport distance because of the strong coulombic interaction[^16^](#_ENREF_16)^,^[^17^](#_ENREF_17). At room temperature, the exciton-depolarization is very strong because intervalley scattering occurs when the exciton energy exceeds a threshold corresponding to twice the energy of the longitudinal acoustic phonon[^12^](#_ENREF_12)^,^[^17^](#_ENREF_17)^,^[^18^](#_ENREF_18). Instead of generating excitonic states, hot-electron injection from the metal through infrared light can realize a long transport length at room temperature[^18^](#_ENREF_18)^,^[^19^](#_ENREF_19), but it has a limited modulation range (10^2^ to 10^3^ ON/OFF ratio) and requires a sophisticated optical source and complicated device structure[^16^](#_ENREF_16).

To realize practical valleytronic applications, it is crucial to use the electric field to generate and control valley[^17^](#_ENREF_17), similar to the conventional charge-based devices. The electrical control of symmetry breaking in bilayer MoS_2_ and graphene provides the potential to achieve valleytronics' electrical operation of valleytronics[^15^](#_ENREF_15)^,^[^20^](#_ENREF_20)^,^[^21^](#_ENREF_21). For single-layer transition metal dichalcogenides, through the injection of spin-polarized carriers and spin–momentum locking, electrical generation of valley polarization has also been realized[^10^](#_ENREF_10). However, these valleytronic works based on the electrical field can only operate under low temperatures.

Due to the robust chiral pumping against thermal perturbation, chiral-anomaly-based valley transport exhibits a long lifetime. The relaxation length can reach 10 to 100 μm at room temperature[^22^](#_ENREF_22)^,^[^23^](#_ENREF_23). The chiral anomaly arises from non-trivial Berry curvature[^24^](#_ENREF_24), which can be modulated through shifting the Fermi level by means of electrical gating[^25^](#_ENREF_25)^,^[^26^](#_ENREF_26). Under ***B*//*E*,** the chiral charge can pump between left-handed Weyl fermion and right-handed Weyl fermion, which leads to the imbalance in the number of particles of each chirality (the so-called chiral anomaly) and the non-equilibrium system[^27^](#_ENREF_27)^,^[^28^](#_ENREF_28)^,^[^29^](#_ENREF_29). **Fig. 1c** presents the chiral charge pumping of left-handed and right-handed chiralities in the lowest Landau levels (N=0). Such a pumping process is compensated by the depletion of valley charge (**Fig. 1d**)**,** described by the inter-valley scattering rate $\tau_{v}^{-1}$.

Experimental signatures such as angle-dependent negative magnetoresistance[^30^](#_ENREF_30), planar Hall effect[^31^](#_ENREF_31), nonlocal transport and magneto-optical Kerr effect experiments[^22^](#_ENREF_22) reveal that chiral anomaly is robust against thermal perturbation. The generated valley can propagate more than 7.5 μm at room temperature[^22^](#_ENREF_22). The room-temperature operation with the electrical generation and control makes chiral-anomaly-based valley transport promising for practical valleytronics.

**Supplementary Note 2. Self-deficiency doping in topological materials results in the high carrier density and a significant shift of the Fermi level**

The presence of defects in materials can induce strong doping and high carrier densities, which dramatically change the position of the Fermi level. For example, the Fermi level of Dirac semimetal Na_3_Bi can be lifted by ~0.4 eV due to self-deficiency induced high carrier densities^[29](#_ENREF_29)^. Lv *et al.* reported that the measured Hall carrier density of 1.5×10^20^ cm^-3^ in Weyl semimetal WTe_1.98_ could upshift the Fermi level by 60~120 meV^[32](#_ENREF_32)^. For the semimetal Cd_3_As_2_, as the carrier densities decrease from 5.2×10^18^ cm^-3^ to 10^17^ cm^-3^, the ***E*_F_** can change from 200 meV to 54 meV^[30](#_ENREF_30)^.

Similar to the self-deficiency-induced shift of ***E*_F_** in topological materials, Te vacancies can increase the hole carrier density (**Supplementary Fig. 1**). Our calculation results (**Supplementary Fig. 2**) are consistent with previous works that Te vacancies-related hole carrier density can shift the ***E*_F_** below the valence band maximum[^1^](#_ENREF_1)^,^[^33^](#_ENREF_33). Specifically, the hole carrier density of 7.4×10^17^ cm^-3^ corresponds to about -0.02 eV below the valence band maximum[^33^](#_ENREF_33). Hall resistance of our Te sample (**Supplementary Fig. 1**) shows a much higher hole density of 1.13×10^19^ cm^-3^ (*n*_2D_ =5.1×10^13^ cm^-2^, 45 nm) at 300 K. According to the relationship between the carrier density and ***E*_F_**[^33^](#_ENREF_33), our high hole density is corresponding to ***E***_F_ position of about -0.18 eV, quite close to the Weyl point of Te (energy difference of only about 20 meV).

**Supplementary Note 3. Extracting the valley resistance**

We extracted the nonlocal valley resistance ($R_{\mathrm{VNL}}$(***B***)) under specific magnetic field (***B***) according to the equation $R_{\mathrm{VNL}}$(***B***)= $R_{\mathrm{NL}}(\boldsymbol{B})$-$R_{\mathrm{ONL}}(\boldsymbol{B})$, where $R_{\mathrm{NL}}(\boldsymbol{B})$ is the tested nonlocal resistance, and $R_{\mathrm{ONL}}(\boldsymbol{B})$ is Ohmic contribution to the measured nonlocal signal. $R_{\mathrm{ONL}}(\boldsymbol{B})$ decays exponentially when electrical contacts are away from the local terminal. Based on the van der Pauw formula[^20^](#_ENREF_20)^,^[^34^](#_ENREF_34), $R_{\mathrm{ONL}}(\boldsymbol{B})$can be described as $R_{\mathrm{ONL}}(\boldsymbol{B})$≈ $R_{L}(\boldsymbol{B})\frac{W}{\pi L}$ $e^{-\pi L/W}$, where *R*_L_ (***B***) is the local resistance, *W* is the channel width, and *L* is the distance between the local terminal and nonlocal terminals. Therefore, we can extract the nonlocal valley resistance by

$R_{\mathrm{VNL}}$(***B***) = $R_{\mathrm{NL}}$(***B***)-$R_{\mathrm{ONL}}$(***B***) =$R_{\mathrm{NL}}$(***B***)-$R_{L}(\boldsymbol{B})\frac{W}{\pi L}$ $e^{-\pi L/W}$

For the width-dependent test, we adopted the device with different widths (**Figure R3b**). x_1_ corresponds to the width of the valley generation region, and x_2_ corresponds to the width of the valley detection region. Local terminal 1-2 and nonlocal terminal 3-4 have the same width x_1_. For the terminal 3-4, we can directly obtain $R_{\mathrm{ONL}}(\boldsymbol{B})=R_{L}(\boldsymbol{B})\frac{W}{\pi L}e^{-\pi L/W}=R_{L}(\boldsymbol{B})\frac{x_{1}}{\pi L}$ $e^{-\pi L/x_{1}}$. As the terminal 5-6 with the width of x_2_ is different from x_1_, $R_{\mathrm{ONL}}\left( B \right)={(R}_{L}(\boldsymbol{B})\frac{x_{2}}{x_{1}})\frac{x_{2}}{\pi L}$ $e^{-\pi L/x_{2}}$, where $R_{L}(\boldsymbol{B})\frac{x_{2}}{x_{1}}$ is the normalized local resistance[^22^](#_ENREF_22).

A dimensionless parameter *k* can qualitatively describe the strength of $R_{\mathrm{VNL}}$ by $R_{\mathrm{VNL}}=kR_{L}\mathbf{(}\boldsymbol{B}\mathbf{)}e^{\boldsymbol{-}L/L_{v}}$, as used in the previous work[^22^](#_ENREF_22). *k*_56_ and *k*_34_ are corresponding coefficient parameters for describing the strength of valley signals in nonlocal terminal 5-6 and 3-4, respectively.

It is noteworthy that for electrode pairs far away from the current source, such as 7 μm in our devices, we can observe $R_{\mathrm{VNL}}$(***B***) ≈ $R_{\mathrm{NL}}$(***B***). The tested nonlocal voltage is almost all from the valley contribution because the signals from conventional charge diffusion (Ohmic contribution) are negligible when nonlocal terminals are away from the drive current.

**Supplementary Note 4. Thickness-dependent carrier densities and *E*_F_**

We conducted the thickness-dependent Hall resistance experiments (**Supplementary Fig. 8a**). The 10-nm-thick Te shows the carrier densities of 1.2×10^12^ cm^-2^. As the thickness increases to 30 nm, the carrier density is 2.8×10^13^ cm^-2^. For the 50-nm-thick sample, the carrier density further reaches 7.3×10^13^ cm^-2^. Thickness-dependent carrier densities are consistent with existing Te works[^2^](#_ENREF_2)^,^[^35^](#_ENREF_35)^,^[^36^](#_ENREF_36).

The carrier densities and ***E***_F_ of Te are dependent on the vacancies. Theoretically, the Te vacancy concentration of ~1.23% can downshift ~0.2 eV below the VBM[^1^](#_ENREF_1). Due to the negative adsorption energy (-0.7 eV) between Te and oxygen[^35^](#_ENREF_35), the forming of the Te-O bond is energetically favourable on the Te surface. Our DFT calculation result shows that the adsorbed oxygen tends to exist in the vicinity of Te vacancy and forms the Te-O bond (**Supplementary Fig. 8b**). The adsorbed oxygen can decrease hole carrier density and upshift the *E*_F_ near the VBM (**Supplementary Fig. 8c**). In this work, we prepared Te samples with different thicknesses by solution method, which usually produces lots of vacancies. Thin Te samples have high surface/bulk ratio and are easy to be passivated by oxygen. Therefore, the thin Te samples have lower hole carrier density and the *E*_F_ is upshifted, away from the Weyl point.

**Supplementary Note 5. Readout power from Ohmic transport contribution**

For the nonlocal device configuration, as most voltage potential difference (*U*_Oh_) uniformly distributes in the local terminal (**Supplementary Figure 7**), the Ohmic-transport power consumption (*P*_Oh_) can be calculated according to *P*_Oh_= ∑*U*_Oh_*I*_Oh_= *I*_Oh_×∑*U*_Oh_≈*I*_constant_*U*_local_, where *I*_Oh_ is the Ohmic current, and *U*_local_ is the voltage difference between local terminal, *I*_constant_ is the constant driving current applied in the local terminal. When we used *I*_constant_=100 nA for testing 8 synaptic valley states, the *U*_local_ is about 40 μV. Then we can calculate *P*_Oh_= *I*_constan_ *U*_local_ =100 nA×40 μV= 4 pW.

Our work exhibits much lower readout power consumption (Ohmic contribution of ~pW and valley contribution of ~fW) than that of synaptic transistors based on Ohmic transport (*e.g.*, ~100 nW based on NdNiO_3_[^3^](#_ENREF_3) and ~1 nW based on Nb_2_O_5_[^8^](#_ENREF_8))_._ Existing non-volatile devices based on Ohmic transport mainly adopt the local terminal readout as the synaptic weight. Relatively high readout current/voltage is necessary for high signal-to-noise ratio, due to side effects of the local terminal (*e.g.*, contact resistance and thermal disturbance on the Ohmic transport).

**Supplementary Note 6. Training procedures of three-layer ANN based on valley transistors**

The constructed neural network is a fully connected feedforward artificial neural network (ANN) with three layers. The ANN consists of 784 input neurons, 100 hidden neurons, and 10 output neurons, where our valley transistors implement the synaptic weights connecting the adjacent neuron layers. 784 input neuron nodes represent the divided 784 pixels in an image, whereas the 10 output neurons correspond to different number classes.

The batch learning method is employed. During each training process, batches of 10 randomly selected images from the training set were used as the inputs for the network. The network was trained based on a hardware friendly backpropagation (BP) algorithm following the Manhattan update rule[^37^](#_ENREF_37). Δ*w* = -∂*E*/∂*w*, *E* is the cross-entropy loss function used to evaluate the classification performance, and *w* is the synaptic weight. When the BP-calculated change of synaptic weight ΔW is positive/negative, one SET/RESET pulse was applied to the valley synaptic transistor. The conductance of the valley synaptic transistor will be updated accordingly.

**Supplementary References**

1 Zhang, N. *et al.* Magnetotransport signatures of Weyl physics and discrete scale invariance in the elemental semiconductor tellurium. *Proc. Natl. Acad. Sci.* **117**, 11337-11343 (2020).

2 Chen, J. *et al.* Topological phase change transistors based on tellurium Weyl semiconductor. *Sci. Adv.* **8**, eabn3837 (2022).

3 Zhang, H.-T. *et al.* Reconfigurable perovskite nickelate electronics for artificial intelligence. *Science* **375**, 533-539 (2022).

4 Zhou, Y. *et al.* Reconfigurable two-WSe_2_-transistor synaptic cell for reinforcement learning. *Adv. Mater.*, e2107754 (2022).

5 Melianas, A. *et al.* Temperature-resilient solid-state organic artificial synapses for neuromorphic computing. *Sci. Adv.* **6**, eabb2958 (2020).

6 Zhou, Y. *et al.* in *2019 IEEE International Electron Devices Meeting (IEDM).* 6.5. 1-6.5. 4 (IEEE).

7 Fuller, E. J. *et al.* Parallel programming of an ionic floating-gate memory array for scalable neuromorphic computing. *Science* **364**, 570-574 (2019).

8 Li, Y. *et al.* Oxide-Based Electrolyte-Gated Transistors for Spatiotemporal Information Processing. *Adv. Mater.* **32**, 2003018 (2020).

9 Yang, C. S. *et al.* All‐Solid‐State Synaptic Transistor with Ultralow Conductance for Neuromorphic Computing. *Adv. Funct. Mater.* **28**, 1804170 (2018).

10 Ye, Y. *et al.* Electrical generation and control of the valley carriers in a monolayer transition metal dichalcogenide. *Nat. Nanotechnol.* **11**, 598-602 (2016).

11 Li, H.-K. *et al.* Valley optomechanics in a monolayer semiconductor. *Nat. Photonics* **13**, 397-401 (2019).

12 Mak, K. F., Xiao, D. & Shan, J. Light–valley interactions in 2D semiconductors. *Nat. Photonics* **12**, 451-460 (2018).

13 Mak, K. F., McGill, K. L., Park, J. & McEuen, P. L. The valley Hall effect in MoS_2_ transistors. *Science* **344**, 1489-1492 (2014).

14 Lee, J., Mak, K. F. & Shan, J. Electrical control of the valley Hall effect in bilayer MoS_2_ transistors. *Nat. Nanotechnol.* **11**, 421-425 (2016).

15 Sui, M. *et al.* Gate-tunable topological valley transport in bilayer graphene. *Nat. Phys.* **11**, 1027-1031 (2015).

16 Zhao, S. *et al.* Valley manipulation in monolayer transition metal dichalcogenides and their hybrid systems: status and challenges. *Rep. Prog. Phys.* **84**, 026401 (2021).

17 Schaibley, J. R. *et al.* Valleytronics in 2D materials. *Nat. Rev. Mater.*  **1**, 1-15 (2016).

18 Li, L. *et al.* Room-temperature valleytronic transistor. *Nat. Nanotechnol.* **15**, 743-749 (2020).

19 Fang, Y., Verre, R., Shao, L., Nordlander, P. & Käll, M. Hot electron generation and cathodoluminescence nanoscopy of chiral split ring resonators. *Nano Lett.* **16**, 5183-5190 (2016).

20 Shimazaki, Y. *et al.* Generation and detection of pure valley current by electrically induced Berry curvature in bilayer graphene. *Nat. Phys.* **11**, 1032-1036 (2015).

21 Wu, S. *et al.* Electrical tuning of valley magnetic moment through symmetry control in bilayer MoS_2_. *Nat. Phys.* **9**, 149-153 (2013).

22 Zhang, C. *et al.* Room-temperature chiral charge pumping in Dirac semimetals. *Nat. Commun.* **8**, 13741 (2017).

23 Parameswaran, S., Grover, T., Abanin, D., Pesin, D. & Vishwanath, A. Probing the chiral anomaly with nonlocal transport in three-dimensional topological semimetals. *Phys. Rev. X* **4**, 031035 (2014).

24 Li, H. *et al.* Negative magnetoresistance in Dirac semimetal Cd_3_As_2_. *Nat. Commun.* **7**, 10301 (2016).

25 Chen, J. W. *et al.* Field-Effect Chiral Anomaly Devices with Dirac Semimetal. *Adv. Funct. Mater.*, 2104192 (2021).

26 Wang, Y. *et al.* Gate-tunable negative longitudinal magnetoresistance in the predicted type-II Weyl semimetal WTe_2_. *Nat. Commun.* **7**, 13142 (2016).

27 Başar, G., Kharzeev, D. E. & Yee, H.-U. Triangle anomaly in Weyl semimetals. *Phys. Rev. B* **89**, 035142 (2014).

28 Zhang, C.-L. *et al.* Signatures of the Adler–Bell–Jackiw chiral anomaly in a Weyl fermion semimetal. *Nat. Commun.* **7**, 10735 (2016).

29 Xiong, J. *et al.* Evidence for the chiral anomaly in the Dirac semimetal Na_3_Bi. *Science* **350**, 413-416 (2015).

30 Li, C. Z. *et al.* Giant negative magnetoresistance induced by the chiral anomaly in individual Cd_3_As_2_ nanowires. *Nat. Commun.* **6**, 10137 (2015).

31 Wu, M. *et al.* Probing the chiral anomaly by planar Hall effect in Dirac semimetal Cd_3_As_2_ nanoplates. *Phys. Rev. B* **98**, 161110 (2018).

32 Lv, Y.-Y. *et al.* Experimental observation of anisotropic Adler-Bell-Jackiw anomaly in type-II Weyl semimetal WTe_1.98_ crystals at the quasiclassical regime. *Phys. Rev. Lett.* **118**, 096603 (2017).

33 Calavalle, F. *et al.* Gate-tuneable and chirality-dependent charge-to-spin conversion in tellurium nanowires. *Nat. Mater.* **21**, 526-532 (2022).

34 Abanin, D. *et al.* Giant nonlocality near the Dirac point in graphene. *Science* **332**, 328-330 (2011).

35 Wang, Y. *et al.* Field-effect transistors made from solution-grown two-dimensional tellurene. *Nat. Electron.* **1**, 228-236 (2018).

36 Lin, Z. *et al.* Two‐Dimensional Tellurene Transistors with Low Contact Resistance and Self‐Aligned Catalytic Thinning Process. *Adv. Electron. Mater.***8**, 2200380 (2022).

37 Prezioso, M. *et al.* Training and operation of an integrated neuromorphic network based on metal-oxide memristors. *Nature* **521**, 61-64 (2015).
